# Design of anti-load perturbation flight trajectory stability controller for agricultural UAV

**DOI:** 10.3389/fpls.2023.1030203

**Published:** 2023-02-03

**Authors:** Xu Wei, Wu XianYu, Liu Jiazhen, Yan Yasheng

**Affiliations:** Marine replenishment department, Naval Logistics Academy, Tianjin, China

**Keywords:** agriculture, UAV, trajectory stability, load perturbation, flight control

## Abstract

The unmanned aerial vehicle(UAV) used in agricultural fields usually has a load, which affects stability of flight trajectory. This problem is a great technical challenge and critical issue which has fascinated many researchers in this area. This research paper presents a flight dynamics model for agricultural UAV under load condition. The robust T-S fuzzy control method is applied in the attitude angle control part, and the traditional PID controller is used in the position loop control part. Results of simulation depict that the flight trajectory of agricultural UAV can reach certain stability when the load is relatively small.

## 1 Introduction

UAVs are widely used in pesticide spraying, sowing, pollination and other agricultural fields. Compared with traditional agricultural machinery, UAV has the advantage of high precision and efficiency (Hu et al., 2022). However UAV is a nonlinear, strongly coupled, under-driven system (Wang and Zhou, 2021). In operating processes, load of UAV changes in real time, which affects speed and accuracy of its flight attitude and trajectory stability (Luo et al., 2020). To improve the flight trajectory stability of UAV against load perturbations for different mission requirements has been a research focus for researchers (Qi and Ping, 2018).

A variety of algorithms are designed to control the flight trajectory stability of UAV,such as PID (Li et al., 2016), linear quadratic regulator (LQR) (Dong et al., 2015), H-infinity (Emam and F Ak.Harian, 2016), sliding mode control (Zhou et al., 2016), back-stepping (Shi and Lin, 2018), and adaptive control (Fan D et al., 2018) and so on. Specifically, A PID intelligent fuzzy control system is designed for spraying operation of agricultural UAV (Zheng and Chen, 2021). An on-line trajectory planning method based on the reinforcement learning is designed for UAV trajectory stability control against swing motion of the slung-payload (Xian et al., 2021). A double closed-loop adaptive control strategy is designed to solve the parameter uncertainty and external disturbance in trajectory tracking process of quad-rotor UAV problem (Gao et al., 2021). The adaptive sliding mode control strategy combined with self-anti-perturbation technique is used, considering the effects on flight performance due to sudden load changes caused by loading/unloading and external perturbation in short-distance transportation (An et al., 2018).

In summary, researches on trajectory control of UAV against load perturbation are remarkable, but the previous researches mostly focus on controlling the suspension load swing perturbation, and some of the research still has improving space of trajectory tracking considering the sudden change of load. Therefore, we designed a different controller for agricultural UAV flight trajectory control against load perturbation. In the attitude angle part, robust T-S fuzzy control method is used. And in the position loop, PID controller method is used. The proposed control method is verified by simulation tests. Load change is simulated by mass change of UAV. Results are showed by outputting UAV flight trajectory tracking image and the changes of roll, pitch and yaw angle.

## 2 Dynamics modelling

**Figure 1:**
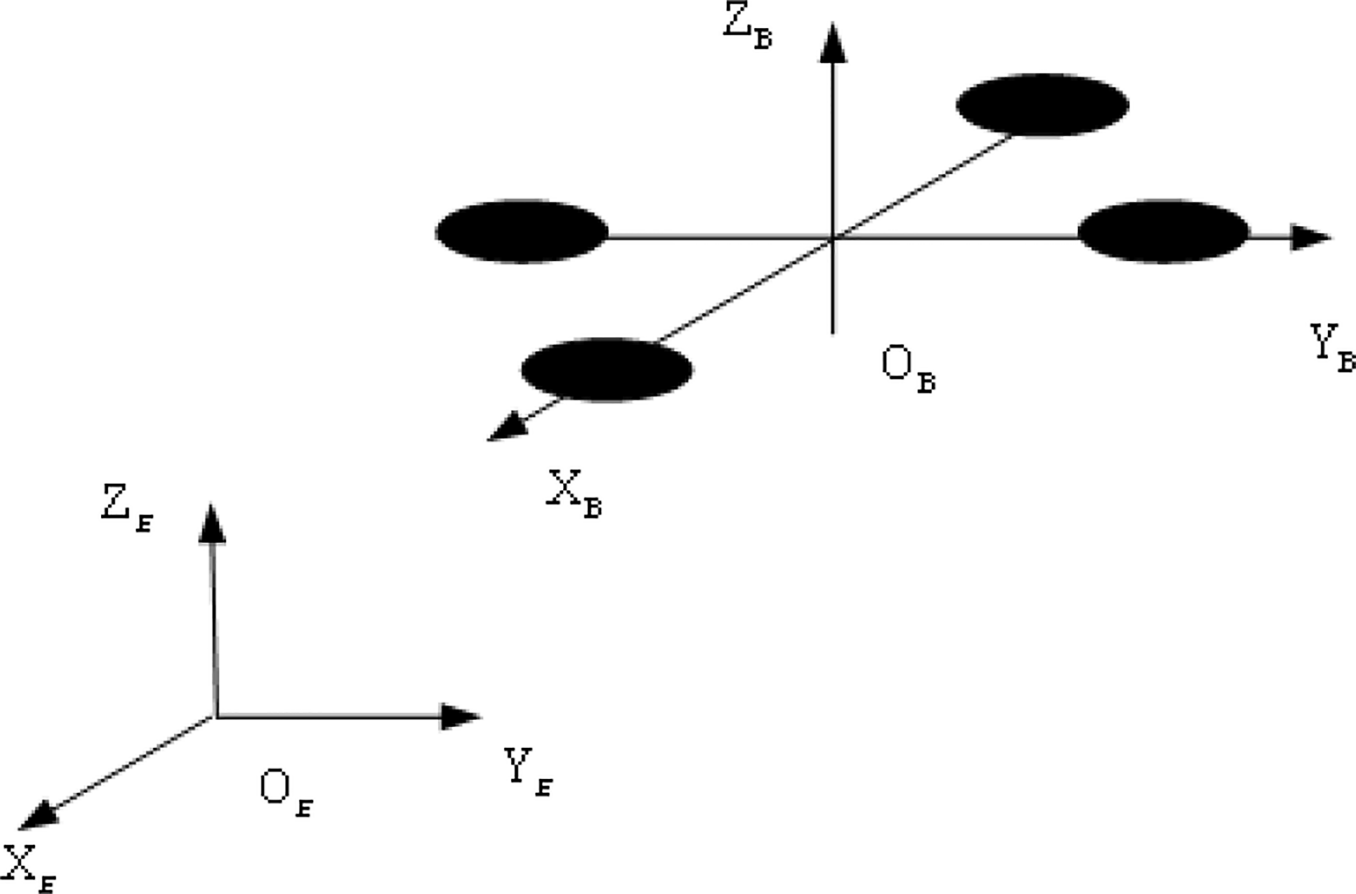
Inertial coordinate system and body coordinate system.

To describe the position and attitude of UAV system, two reference coordinate systems are established: inertial coordinate system{E}, and body coordinate system{B} as shown in **Figure 1**.

1) inertial coordinate system{E},(O_E_,X_E_,Y_E_,Z_E_). It is fixed to the Earth and follows the right-hand rule, forming a right-handed, right-angle coordinate system in the order of “North, West and Sky”, i.e. the axis X_E_ points to the North, the axis Y_E_ points to the West, and the axis Z_E_ is perpendicular to the ground and back from the center of the Earth. O_E_ is the origin.

2) body coordinate system{B},(O_B_,X_B_,Y_B_,Z_B_). It is a right-angle coordinate system solidly connected to UAV, following right-hand rule. O_B_ is the origin, located at the center of UAV gravity. The axis X_B_ in symmetry plane of UAV, parallels to UAV design axis and directs to the nose. The axis Y_B_ is perpendicular to symmetry plane of UAV and points to the left of the fuselage. The axis Z_B_ is in symmetry plane of UAV, perpendicular to plane X_B_-Y_B_ and points above the fuselage.

The Euler angle $\text{Θ}={\left[\begin{array}{ccc} \phi & \text{θ} & \text{ψ} \end{array}\right]}^{\text{T}}$ is used to represent the attitude angle vector of UAV in the inertial system{E}, which is defined as follows.

*ϕ* : Roll angle means rotation angle of UAV body around axis X_B_, with positive rotation to the right.
θ : Pitch angle means rotation angle of UAV body around axis Y_B_, with positive rotation upward.
ψ : Yaw angle refers to rotation angle of UAV body around axis Z_B_, with positive rotation to the right.

In the beginning, the above two coordinate systems coincide. Each coordinate vector of {B} can be converted to corresponding coordinate vector of {E} by coordinate transformation and vice versa. Rotation matrix is described by the parameters in Euler angles $\text{Θ}={\left[\begin{array}{ccc} \phi & \text{θ} & \text{ψ} \end{array}\right]}^{\text{T}}$ . The specific derivation process is as follows.

**Figure 2:**
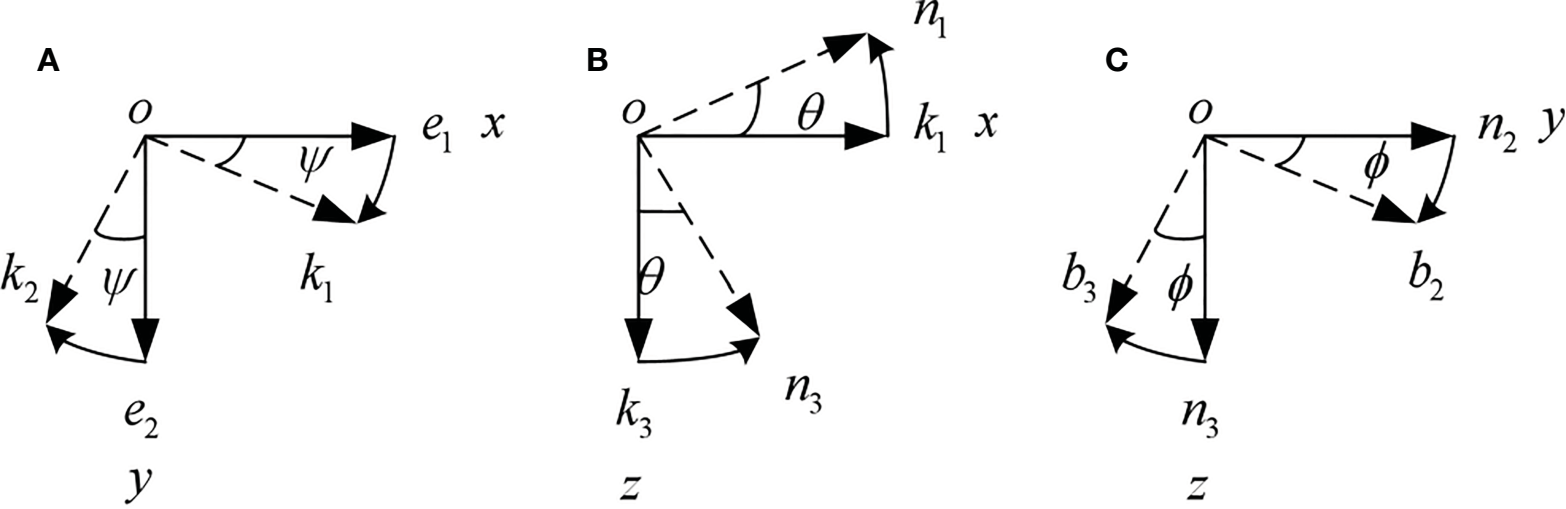
Coordinate transformation. **(A)** Rotating around Z-axis. **(B)** Rotating around Y-axis. **(C)** Rotating around X-axis.


When rotating around Z-axis in inertial coordinate system, as shown in **Figure 2A**, the transformation equation is

(1) $$\{\begin{array}{c} x=\cos(\text{ψ})x+\sin\left(\text{ψ}\right)y\\ y=-\sin\left(\text{ψ}\right)x+\cos(\text{ψ})y\\ z=z \end{array}$$

The conversion matrix is


(2) $$\text{R}\left(\text{z},\text{ψ}\right)=\left[\begin{array}{ccc} \cos\text{ψ} & \sin\text{ψ} & 0\\ -\sin\text{ψ} & \cos\text{ψ} & 0\\ 0 & 0 & 1 \end{array}\right]$$

When rotating around Y-axis, as shown in **Figure 2B**, the transformation equation is


(3) $$\{\begin{array}{c} x=\cos(\text{θ})x+\sin\left(\text{θ}\right)y\\ y=y\\ z=\sin\left(\text{θ}\right)x+\cos(\text{θ})z \end{array}$$

The conversion matrix is


(4) $$\text{R}\left(\text{y},\text{θ}\right)=\left[\begin{array}{ccc} \cos\text{θ} & 0 & -\sin\text{θ}\\ 0 & 1 & 0\\ \sin\text{θ} & 0 & \cos\text{θ} \end{array}\right]$$

When rotating around X-axis, as shown in **Figure 2C**, the transformation equation is


(5) $$\{\begin{array}{c} x=x\\ y=cos\left(\phi \right)y+\sin\left(\phi \right)z\\ z=-\sin\left(\phi \right)y+cos\left(\phi \right)z \end{array}$$

The conversion matrix is


(6) $$\text{R}\left(x,\phi \right)=\left[\begin{array}{ccc} 1 & 0 & 0\\ 0 & \cos\phi & \sin\phi \\ 0 & -\sin\phi & \cos\phi \end{array}\right]$$

Transformation from {E} to{B} can be achieved by following yaw angle, then pitch angle, and then roll angle. The transformation matrix is equal to chain-multiplication of the matrices obtained from sequential rotations, i.e.


(7) $${\text{R}}_{\text{E}}^{\text{B}}=\text{R}\left(x,\phi \right)\text{R}\left(y,\text{θ}\right)\text{R}\left(z,\text{ψ}\right)$$

Substituting equations (2), (4), and (6), into equation (7), rotation matrix from {E} to {B} is obtained: (8); similarly, we can get rotation matrix from {B} to {E}, which is an orthogonal array: (9)


(8) $${\text{R}}_{\text{E}}^{\text{B}}=\left[\begin{array}{ccc} \cos\text{θ}\cos\text{ψ} & \cos\text{θ}\sin\text{ψ} & 0\\ \sin\phi \sin\text{θ}\cos\text{ψ}-\cos\phi \sin\text{ψ} & \sin\phi \sin\text{θ}\sin\text{ψ}+\cos\phi \cos\text{ψ} & \sin\phi \cos\text{θ}\\ \cos\phi \sin\text{θ}\cos\text{ψ}+\sin\text{θ}\sin\text{ψ} & \cos\phi \sin\text{θ}\sin\text{ψ}-\sin\phi \cos\text{ψ} & \cos\phi \cos\text{θ} \end{array}\right]$$

(9) $${\text{R}}_{\text{B}}^{\text{E}}=\left[\begin{array}{ccc} \cos\text{θ}\cos\text{ψ} & \sin\phi \sin\text{θ}\cos\text{ψ}-\cos\phi \sin\text{ψ} & \cos\phi \sin\text{θ}\cos\text{ψ}+\sin\text{θ}\sin\text{ψ}\\ \cos\text{θ}\sin\text{ψ} & \sin\phi \sin\text{θ}\sin\text{ψ}+\cos\phi \cos\text{ψ} & \cos\phi \sin\text{θ}\sin\text{ψ}-\sin\phi \cos\text{ψ}\\ -\sin\text{θ} & \sin\phi \cos\text{θ} & \cos\phi \cos\text{θ} \end{array}\right]$$

According to ${\text{ν}}_{\text{E}}={\text{R}}_{\text{B}}^{\text{E}}{\text{V}}_{\text{B}}$ , where velocity under body coordinate system ${\text{V}}_{\text{B}}=\left({\dot{X}}_{\text{B}},{\dot{Y}}_{\text{B}},{\dot{Z}}_{\text{B}}\right)$ , velocity under inertial coordinate system ${\text{V}}_{\text{E}}=\left({\dot{X}}_{\text{E}},{\dot{Y}}_{\text{E}},{\dot{Z}}_{\text{E}}\right)$ . Position state equations can be solved.

Similarly, angular velocity transformation relation from {B} to {E} can be obtained, taking X-axis as an example.


(10) $$\omega^{\text{E}}=\left[\begin{array}{c} \omega_{x}\\ \omega_{y}\\ \omega_{z} \end{array}\right]=\text{R}\left(x,\phi \right)\text{R}\left(y,\text{θ}\right)\left[\begin{array}{c} 0\\ 0\\ \dot{\psi } \end{array}\right]+\text{R}\left(x,\phi \right)\left[\begin{array}{c} 0\\ \dot{\theta }\\ 0 \end{array}\right]+\left[\begin{array}{c} \dot{\phi }\\ 0\\ 0 \end{array}\right]=\left[\begin{array}{ccc} 1 & 0 & -\sin\theta \\ 0 & \cos\phi & \cos\text{θ}\sin\phi \\ 0 & -\sin\phi & \cos\phi \cos\text{θ} \end{array}\right]\left[\begin{array}{c} \dot{\phi }\\ \dot{\theta }\\ \dot{\psi } \end{array}\right]$$

Transforming the formula(10) gives angular velocity transformation matrix from {E} to {B}.


(11) $$\left[\begin{array}{c} \dot{\phi }\\ \dot{\theta }\\ \dot{\psi } \end{array}\right]=\left[\begin{array}{ccc} 1 & \tan\theta \sin\phi & \tan\theta \cos\phi \\ 0 & \cos\phi & -\sin\phi \\ 0 & \sin\phi /\cos\text{θ} & \cos\phi /\cos\text{θ} \end{array}\right]$$

**Figure 3:**
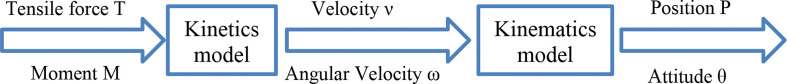
A rigid body model of UAV flight control.

The purpose of establishing UAV dynamics model is to analyze the variations of UAV trajectory under external forces and moments. The input of kinetics model is external forces and moments provided by the propeller. The output of kinetics model is velocity and angular velocity, which is input of the kinematics model. Then output of the kinematic model is position and attitude angle. The relationships are as shown in **Figure 3**.

### 2.1 Kinetic model

According to Newton-Euler Equations, motion of a rigid body equals translation of centroid plus rotation around centroid. Translation of centroid is described by Newton’s second law, i.e. $F=m\frac{dv}{dt}$ , where *F* is resultant external force; *v* is velocity. Rotation round centroid is described by Euler equation, i.e. $M=J\dot{\omega }+\omega \times J\omega$ , where *M* is resultant external moment; *J* is 3×3 inertia matrix, and ω is angular velocity.

Then position dynamics model is ${\dot{\upsilon }}^{E}=\frac{f^{B}}{m}-g^{E}$ , where the letter *E* indicates vectors in {E} and the letter *B* indicates vectors in {B}. For convenience, force *f* is converted to Earth coordinate system by left multiplying rotation matrix ${\text{R}}_{\text{B}}^{\text{E}}:{\dot{\upsilon }}^{e}={\text{R}}_{\text{B}}^{\text{E}}\frac{f^{B}}{m}-g^{E}$ .

**Figure 4:**
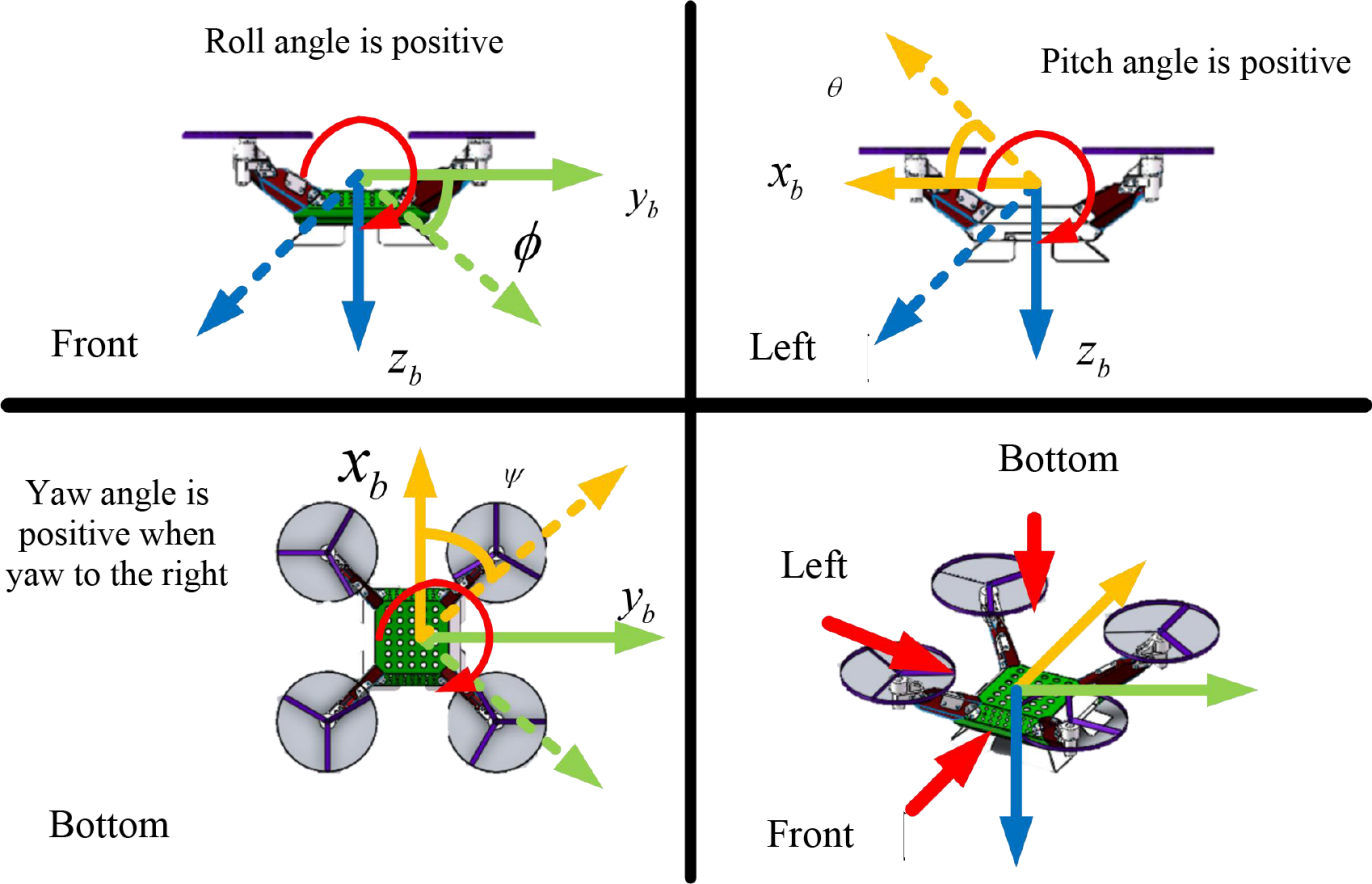
Attitude angle of agricultural UAV.

φ, θ, ψ respectively indicate roll angle of X-axis rotation, pitch angle of Y-axis rotation, and yaw angle of Z-axis rotation, i.e., the Euler angle, as shown in **Figure 4**.

Expanding the equation and substituting ${\text{R}}_{\text{B}}^{\text{E}}$ into it can be obtained as


(12) $$\{\begin{array}{c} {\dot{\upsilon }}_{x}=\frac{f}{m}\left(\cos\text{ψ}\sin\text{θ}\cos\phi +\sin\text{ψ}\sin\phi \right)\\ {\dot{\upsilon }}_{y}=\frac{f}{m}\left(\sin\text{ψ}\sin\text{θ}\cos\phi -\sin\phi \cos\text{ψ}\right)\\ {\dot{\upsilon }}_{z}=\frac{f}{m}\left(\cos\text{θ}\cos\text{ψ}\right)-g \end{array}$$

This yields the equations regarding combined external force and velocity——the position kinetic model. Then we talk about the equations regarding combined moment and angular velocity——the attitude kinetic model.

The angular velocity equation is that the body makes a rotational motion around the axis under the combined external moment, so that the UAV attitude Euler angle $\text{Θ}={\left[\begin{array}{ccc} \phi & \text{θ} & \text{ψ} \end{array}\right]}^{\text{T}}$ changes. Moments action that UAV is subjected to when flying mainly includes: aerodynamic effects, inertial counter-torsional moments and gyroscopic moments.

For moment analysis of UAV, the angular motion equation can be obtained according to Euler’s equation as $J{\dot{\omega }}^{B}+\omega^{B}\times J\omega^{B}=G_{a}+\tau$ , where, *ω* ^*B*^ denotes angular velocity in {B}; *G*_*a*_ denotes the gyroscopic moment; *τ* denotes the counter-torsional moment generated by propeller on the body axis, including roll moment *τ*_*x*_ around axis O*_b_x_b_*, pitch moment *τ*_*y*_ around axis O*_b_y_b_*, and the yaw moment *τ*_*z*_ around axis O*_b_z_b_*.

Regarding ω^B^ , for convenience, the three components ω_*xb*_ , ω_*yb*_ , ω_*zb*_ are denoted by *p*, *q*, and *r* respectively, i.e.


(13) $$\omega^{\text{B}}=\left[\begin{array}{c} \omega_{xb}\\ \omega_{yb}\\ \omega_{zb} \end{array}\right]=\left[\begin{array}{c} p\\ q\\ r \end{array}\right]$$

Inertia matrix of UAV in the body coordinate system consists of nine components, denoted as


(14) $$\text{J}=\left[\begin{array}{ccc} {\text{I}}_{xx} & -{\text{I}}_{xy} & -{\text{I}}_{xz}\\ -{\text{I}}_{yx} & {\text{I}}_{\text{yy}} & -{\text{I}}_{yz}\\ -{\text{I}}_{zx} & -{\text{I}}_{zy} & {\text{I}}_{zz} \end{array}\right]$$

where I_*xx*_ , I_yy_ , I_zz_ are moments of inertia, while I_*xy*_ , I_*xz*_ , I_*yz*_ , etc. are products of inertia.


(15) $$\{\begin{array}{c} {\text{I}}_{xx}=\sum m_{i}\left(y_{i}^{2}+z_{i}^{2}\right)=\int \left(y^{2}+z^{2}\right)dm\\ {\text{I}}_{yy}=\sum m_{i}\left(z_{i}^{2}+x_{i}^{2}\right)=\int \left(z^{2}+x^{2}\right)dm\\ {\text{I}}_{\text{zz}}=\sum m_{i}\left(x_{i}^{2}+y_{i}^{2}\right)=\int \left(x^{2}+y^{2}\right)dm \end{array}$$

(16) $$\{\begin{array}{c} {\text{I}}_{xy}={\text{I}}_{yx}=\sum m_{i}x_{i}y_{i}=\int \left(xy\right)dm\\ {\text{I}}_{xz}={\text{I}}_{zx}=\sum m_{i}x_{i}z_{i}=\int \left(xz\right)dm\\ {\text{I}}_{\text{yz}}={\text{I}}_{\text{zy}}=\sum m_{i}y_{i}z_{i}=\int \left(yz\right)dm \end{array}$$

Since the UAV structure is uniformly symmetric and the origin of body coordinate system coincides with the centroid. The integral of an odd function in the symmetry region is zero, thus the inertia matrix is


(17) $$\text{J}=\left[\begin{array}{ccc} {\text{I}}_{xx} & 0 & 0\\ 0 & {\text{I}}_{\text{yy}} & 0\\ 0 & 0 & {\text{I}}_{\text{zz}} \end{array}\right]$$

The second term on the left side of Euler’s equation can be written as


(18) $$\omega^{B}+J\omega^{B}=\left[\begin{array}{ccc} i & j & k\\ p & q & r\\ {\text{I}}_{xx}\text{p} & {\text{I}}_{\text{yy}}\text{q} & {\text{I}}_{\text{zz}}\text{r} \end{array}\right]=\left[\begin{array}{c} qr\left({\text{I}}_{\text{zz}}-{\text{I}}_{\text{yy}}\right)\\ pr\left({\text{I}}_{\text{xx}}-{\text{I}}_{\text{zz}}\right)\\ pq\left({\text{I}}_{\text{xx}}-{\text{I}}_{\text{yy}}\right) \end{array}\right]$$

Gyroscopic torque *G*_*a*_ : When a motor rotates at high speed, it is equivalent to a gyroscope. A gyroscope spinning at high speed is a very stable individual with the ability to keep its axial direction unchanged. Therefore if an external force tries to change the direction of the gyroscopic axis, a gyroscopic moment will be generated to resist this change.

The equation for the gyroscopic moment is given by *G*_*a*_=*J*_*RP*_Ω×*ϖ* , where Ω is the propeller angular velocity vector, *ϖ* is the rotational angular velocity of motor rotor shaft, and we consider the rotor shaft angular velocity to be equal to the body angular velocity. Gyroscopic moment of the rotor assembly k (with motor and propeller) is *G*_*a*,*k*_=*J*_*RP*_Ω_*k*_ *b*_3_×*ϖ* , where *b*_3_ is direction of the propeller angular velocity. Because the propeller only has angular velocity in Z-axis direction in {B}, $b_{3}={\left[\begin{array}{ccc} 0 & 0 & 1 \end{array}\right]}^{T}$ . *J*_*RP*_ is total rotational inertia of the motor rotor and the propeller. *Ω*_*k*_ is the *k*th propeller angular velocity, according to the right-hand rule, the clockwise rotation around z-axis is positive. When *k*=1, the propeller speed is negative, *Ω*_*k*_=−*ϖ*_1_ , the speed of the remaining propellers and so on.


(19) $$b_{3}\times \varpi =-\left(\varpi \times \right)b_{3}=\left[\begin{array}{ccc} 0 & -r & q\\ r & 0 & -p\\ -q & p & 0 \end{array}\right]\left[\begin{array}{c} 0\\ 0\\ 1 \end{array}\right]=\left[\begin{array}{c} q\\ -p\\ 0 \end{array}\right]$$

“× “ denotes the cross operator.

Formula for *G*_*a*_ can be obtained as


(19) $$G_{a}=\left[\begin{array}{c} G_{a,\phi }\\ G_{a,\theta }\\ G_{a,\psi } \end{array}\right]=-{\text{J}}_{\text{RP}}\omega_{k}\left[\begin{array}{c} q\\ -p\\ 0 \end{array}\right]=\left[\begin{array}{c} -q{\text{J}}_{\text{RP}}\left(\varpi_{1}-\varpi_{2}+\varpi_{3}-\varpi_{4}\right)\\ p{\text{J}}_{\text{RP}}\left(-\varpi_{1}+\varpi_{2}-\varpi_{3}+\varpi_{4}\right)\\ 0 \end{array}\right]$$

Where *ϖ*_1_,*ϖ*_2_,*ϖ*_3_,*ϖ*_4_ indicates speed of propellers 1, 2, 3, and 4.

Organizing the above equations, we have


(20) $$\left[\begin{array}{c} I_{xx}\dot{p}\\ I_{yy}\dot{q}\\ I_{zz}\dot{r} \end{array}\right]=\left[\begin{array}{c} qr\left({\text{I}}_{\text{yy}}-{\text{I}}_{\text{zz}}\right)\\ pr\left({\text{I}}_{\text{zz}}-{\text{I}}_{\text{xx}}\right)\\ pq\left({\text{I}}_{\text{xx}}-{\text{I}}_{\text{yy}}\right) \end{array}\right]=\left[\begin{array}{c} -q{\text{J}}_{\text{RP}}\left(\varpi_{1}-\varpi_{2}+\varpi_{3}-\varpi_{4}\right)\\ p{\text{J}}_{\text{RP}}\left(-\varpi_{1}+\varpi_{2}-\varpi_{3}+\varpi_{4}\right)\\ 0 \end{array}\right]+\left[\begin{array}{c} \tau_{x}\\ \tau_{y}\\ \tau_{z} \end{array}\right]$$

### 2.2 Kinematic model

The position equation for UAV is ${\dot{p}}^{E}=v^{E}$ , where ${\dot{p}}^{E}={\left[\begin{array}{ccc} x & y & z \end{array}\right]}^{T}$ is coordinate position in {E}, and the expansion yields: ${\left[\begin{array}{ccc} \dot{x} & \dot{y} & \dot{z} \end{array}\right]}^{T}={\left[\begin{array}{ccc} v_{x} & v_{y} & v_{z} \end{array}\right]}^{T}$ .

Then we use Euler’s angle to express the attitude. The change rate of attitude angle is related to body rotational angular velocity as follows.


(21) $$\dot{\Theta }=\left[\begin{array}{c} \dot{\phi }\\ \dot{\theta }\\ \dot{\psi } \end{array}\right]=\text{W}\cdot \omega^{\text{B}}=\left[\begin{array}{ccc} 1 & \tan\theta \sin\phi & \tan\theta \cos\phi \\ 0 & \cos\phi & -\sin\phi \\ 0 & \sin\phi /\cos\text{θ} & \cos\phi /\cos\text{θ} \end{array}\right]\left[\begin{array}{c} p\\ q\\ r \end{array}\right]$$

Where $\text{Θ}={\left[\begin{array}{ccc} \dot{\phi } & \dot{\theta } & \dot{\psi } \end{array}\right]}^{\text{T}}$ , $\omega^{\text{B}}={\left[\begin{array}{ccc} \omega_{xb} & \omega_{yb} & \omega_{zb} \end{array}\right]}^{\text{T}}={\left[\begin{array}{ccc} p & q & r \end{array}\right]}^{\text{T}}$ When small perturbations happen, i.e., provided that variation of each angle is small, and change rate of attitude angle is approximately equal to the body rotational angular velocity, then we have


(22) $$\left[\begin{array}{c} \dot{\phi }\\ \dot{\theta }\\ \dot{\psi } \end{array}\right]=\left[\begin{array}{c} p\\ q\\ r \end{array}\right]$$

The rigid-body flight control model of UAV consists of the kinetic model and the kinematic model. According to the above equations, there are


(23) $$\{\begin{array}{c} {\ddot{x}}_{x}=\frac{f\left(c\phi s\theta c\psi +s\phi s\psi \right)}{m}\\ \ddot{y}=\frac{c\phi s\theta c\psi -s\phi s\psi }{m}{\text{U}}_{1}\\ \ddot{z}=\frac{c\phi c\theta }{m}{\text{U}}_{1}-g \end{array}$$

(24) $$\{\begin{array}{c} \ddot{\phi }=\frac{I{\text{U}}_{2}+\dot{\theta }\dot{\psi }\left({\text{I}}_{\text{yy}}-{\text{I}}_{\text{zz}}\right)}{{\text{I}}_{\text{xx}}}\\ \ddot{\theta }=\frac{I{\text{U}}_{3}+\dot{\phi }\dot{\psi }\left({\text{I}}_{\text{zz}}-{\text{I}}_{\text{xx}}\right)}{{\text{I}}_{\text{yy}}}\\ \ddot{\psi }=\frac{I{\text{U}}_{4}+\dot{\phi }\dot{\psi }\left({\text{I}}_{\text{zz}}-{\text{I}}_{\text{xx}}\right)}{{\text{I}}_{\text{zz}}} \end{array}$$

The following equations can be obtained.


(25) $$\{\begin{array}{c} {\dot{p}}^{E}=v^{E}\\ {\dot{v}}^{E}=g{\text{e}}_{3}-\frac{f}{m}R{\text{e}}_{3}\\ \dot{\Theta }=W\cdot \omega^{\text{B}}\\ J\cdot \omega^{\text{B}}=-\omega^{\text{B}}\times \left(J\cdot \omega^{\text{B}}\right)+{\text{G}}_{\text{a}}+\tau \end{array}$$

## 3 Controller design

### 3.1 Attitude angle control

The robust T-S fuzzy control method is proposed in attitude angle control part, which demands a linear expression of the nonlinear model. Therefore, we need to build the robust T-S fuzzy control model. A robust T-S fuzzy control system is a linear time-varying system whose matrix depends on a time-varying parameter vector that varies within a known interval.

In order to represent the dynamics of UAV with a set of local linear models, a nonlinear representation of the sector based on the weight and affiliation function is applied to attitude angle. Assuming that the aerodynamic effects including blade grinding, rotor inertia or drag force, are negligible, the attitude angle can be rewritten as a multiple linearized model based on the operating points. The operating points are mainly determined by the roll rate, pitch rate and yaw rate (i.e., the first order derivatives of the corresponding angles), i.e. $\text{P}=\left[\begin{array}{ccc} \dot{\phi } & \dot{\theta } & \dot{\psi } \end{array}\right]$ . The dynamics linearized model of the attitude angle is


(26) $$\dot{X}={\text{A}}_{\text{i}}{\text{X}}_{\text{E}}+\text{BU}$$

Where $X_{E}={\left[e_{\phi }\;\dot{e_{\phi }}\;e_{\theta }\;\dot{e_{\theta }}\;e_{\psi }\;\dot{e_{\Psi }}\;q_{1}\;q_{2}\;q_{3}\right]}^{T}$ , $\text{U}={\left[\begin{array}{ccc} {\text{U}}_{2} & {\text{U}}_{3} & {\text{U}}_{4} \end{array}\right]}^{\text{T}}$ , $\dot{q}={\left[\begin{array}{ccc} {\dot{q}}_{1} & {\dot{q}}_{2} & {\dot{q}}_{2} \end{array}\right]}^{\text{T}}={\left[\begin{array}{ccc} {\text{e}}_{\phi } & {\text{e}}_{\text{θ}} & {\text{e}}_{\text{ψ}} \end{array}\right]}^{\text{T}}$ , *θ* ^*ref*^ , *ψ* ^*ref*^ are given by inverse solution of the position control loop, *ψ* ^*ref*^ =0;*i=*1,2,.,*r*, *r* denotes the number of control rates.

The concept of sector nonlinearity is applied to this T-S fuzzy model. If the *j*th operating point is restricted to α_max_ and α_min_, the weighting function is expressed as follows


(27) $$\omega_{1}^{\text{j}}=1-\omega_{0}^{\text{j}}=1-\frac{{\text{α}}_{\text{max}}-{\text{P}}_{\text{j}}}{{\text{α}}_{\text{max}}-{\text{α}}_{\text{min}}}$$

Then an affiliation function based on the weighting function is defined as


(28) $$h_{i}=\prod_{j=1}^{p}\omega_{i_{j}}^{j}$$

where *p* is the number of operator points and *i*=1,······,2*p* , *p=*3, then *r*=2*p*=8. Defining *h_i_* >0, *P_i_* is restricted to α_max_ and α_min_. *P*_*i*_∈[−2,2],rad/s . According to the above equation, we can obtain $\sum_{\text{i}=1}^{\text{r}}h_{i}=1$ . Using this affiliation function, the linearized model of attitude angle dynamics can be expressed as


(29) $$\dot{X}=\sum_{\text{i}=1}^{\text{r}}h_{i}K_{i}X_{E}+\text{BU}$$

where *K_i_* is the controller gain calculated by the robust T-S fuzzy system, i.e., every possible upper and lower bound arrangement of the components in P.

The eight rules of the robust T-S fuzzy system are shown in **Table 1**.

**Table 1:** T-S control rate.

parameter	$\dot{\phi }$	$\dot{\theta }$	$\dot{\psi }$
**h_1_**	Min	Min	Min
**h_2_**	Min	Min	Max
**h_3_**	Min	Max	Min
**h_4_**	Min	Max	Max
**h_5_**	Max	Min	Min
**h_6_**	Max	Min	Max
**h_7_**	Max	Max	Min
**h_8_**	Max	Max	Max

To design robust T-S fuzzy system controller gain, the robust T-S fuzzy system optimal control problem is expressed as an optimization problem with LMI constraints.

Given the LMI parameters Q=QT≥0,R=RT>0, the optimal performance bound μ>0 (such that J(x, u)<μ), and the decay rate η>0. Then, controller gain *K*_*i*_ calculated by the robust T-S fuzzy system is obtained by solving P∈S^9×9^ , W_i_∈S^3×9^ , such that μ is minimized while meeting the following LMIs:


(30) $$\{\begin{array}{c} \left({\text{A}}_{\text{i}}P+B{\text{W}}_{\text{i}}\right)+{\left({\text{A}}_{\text{i}}\text{P}+{\text{BW}}_{\text{i}}\right)}^{\text{T}}+2\eta P\leq 0\\ trace\left({\text{Q}}^{\frac{1}{2}}P{\left({\text{Q}}^{\frac{1}{2}}\right)}^{\text{T}}\right)+trace\left(Y\right)\leq \mu \\ \left|\begin{array}{cc} -\text{Y} & {\text{R}}^{\frac{1}{2}}{\text{W}}_{\text{i}}\\ {\left({\text{R}}^{\frac{1}{2}}{\text{W}}_{\text{i}}\right)}^{\text{T}} & -\text{P} \end{array}\right|\leq 0 \end{array}$$

Then *K*_*i*_=−W_i_P^−1^ , it guarantees global stability while providing the required transient behavior.

Where


$$\text{R}=[\begin{array}{ccc} 2 & 0 & 0\\ 0 & 0.5 & 0\\ 0 & 0 & 0.5 \end{array}],\text{Q}={\text{I}}_{9}.$$

Based on the above LMI pole configuration method and T-S fuzzy system, the attitude angle robust T-S fuzzy controller is designed, and we get the attitude angle control law as


(31) $$\text{U}=\left[\begin{array}{c} {\text{U}}_{2}\\ {\text{U}}_{3}\\ {\text{U}}_{4} \end{array}\right]=\sum_{\text{i}=1}^{\text{r}}h_{i}K_{i}X_{E}$$

### 3.2 Position loop control

For the position loop control part, a PID controller is used, with the error defined as


(32) $$e_{i}=X_{d}=X_{i}-X_{i}^{ref}$$

Where $X=\left[\begin{array}{ccc} x & y & z \end{array}\right]$ , denotes the real-time coordinates and attitude angle values of UAV obtained by the sensors, and

$X^{ref}=\left[\begin{array}{ccc} x^{ref} & y^{ref} & z^{ref} \end{array}\right]$ denotes the desired coordinates of UAV. The desired coordinates of UAV are given by its desired trajectory. The desired attitude angle *θ* ^*ref*^ , *ψ* ^*ref*^ are given by the following equations.


(33) $$\{\begin{array}{c} \phi^{ref}=arcsin\left(\frac{m\left(U_{x}sin\left(\Psi \right)-U_{y}sin\left(\Psi \right)\right)}{U_{1}}\right)\\ \theta^{ref}=arcsin\left(\frac{mU_{x}-U_{1}sin\left(\phi \right)cos\left(\Psi \right))}{U_{1}cos\left(\phi \right)cos\left(\Psi \right)}\right)\\ U_{x}=cos\left(\phi \right)sin\left(\theta \right)cos\left(\psi \right)+sin\left(\phi \right)sin\left(\Psi \right)\\ U_{y}=cos\left(\phi \right)sin\left(\theta \right)cos\left(\psi \right)-sin\left(\phi \right)sin\left(\Psi \right) \end{array}$$

The error matrix of the position loop is defined as $e=\left[\begin{array}{ccc} x_{d} & y_{d} & z_{d} \end{array}\right]$ . According to the PID control law, controller for error handling can be written as


(34) $$u=k_{p}e+k_{i}\int edt+k_{d}\frac{de}{dt}$$

Then the control law of position loop is $U_{1}=\sqrt{U_{x}^{2}+U_{y}^{2}+{\left(U_{z}-g\right)}^{2}}$ , *U*_*x*_ , *U*_*y*_ , *U*_*z*_ is given by


(34) $$\left[\begin{array}{c} U_{x}\\ U_{y}\\ U_{z} \end{array}\right]=K_{PID}{\left[x_{d}\;\dot{x_{d}}\;y_{d}\;\dot{y_{d}}\;z_{d}\;{\dot{z}}_{d}\;\int x_{d}\int y_{d}\int z_{d}\right]}^{\text{T}}$$

*K*_*PID*_ is the control parameter.

## 4 Simulation verification

The simulation experiments are based on UAV parameters as shown in **Table 2**. Considering flight trajectory stability of UAV under load perturbation, load perturbation is simulated by mass change of UAV.

**Table 2:** UAV parameters (Shen et al., 2021; Lotufo et al., 2020).

symbol	Instructions	value
m	Mass	1.79kg
I_xx_	X-axis moment of inertia	0.03 kg/m^2^
I_yy_	Y-axis moment of inertia	0.03 kg/m^2^
I_zz_	Z-axis moment of inertia	
l	Distance from rotor to center point	0.2m
C	Pneumatic coefficient	0.221
S_x_	X-axis windward area	0.1 m^2^
S_y_	Y-axis windward area	0.1 m^2^
S_z_	Z-axis windward area	0.2m^2^
ρ	Air Density	1.293 Kg/m

### 4.1 No change in UAV mass

**Figure 5:**
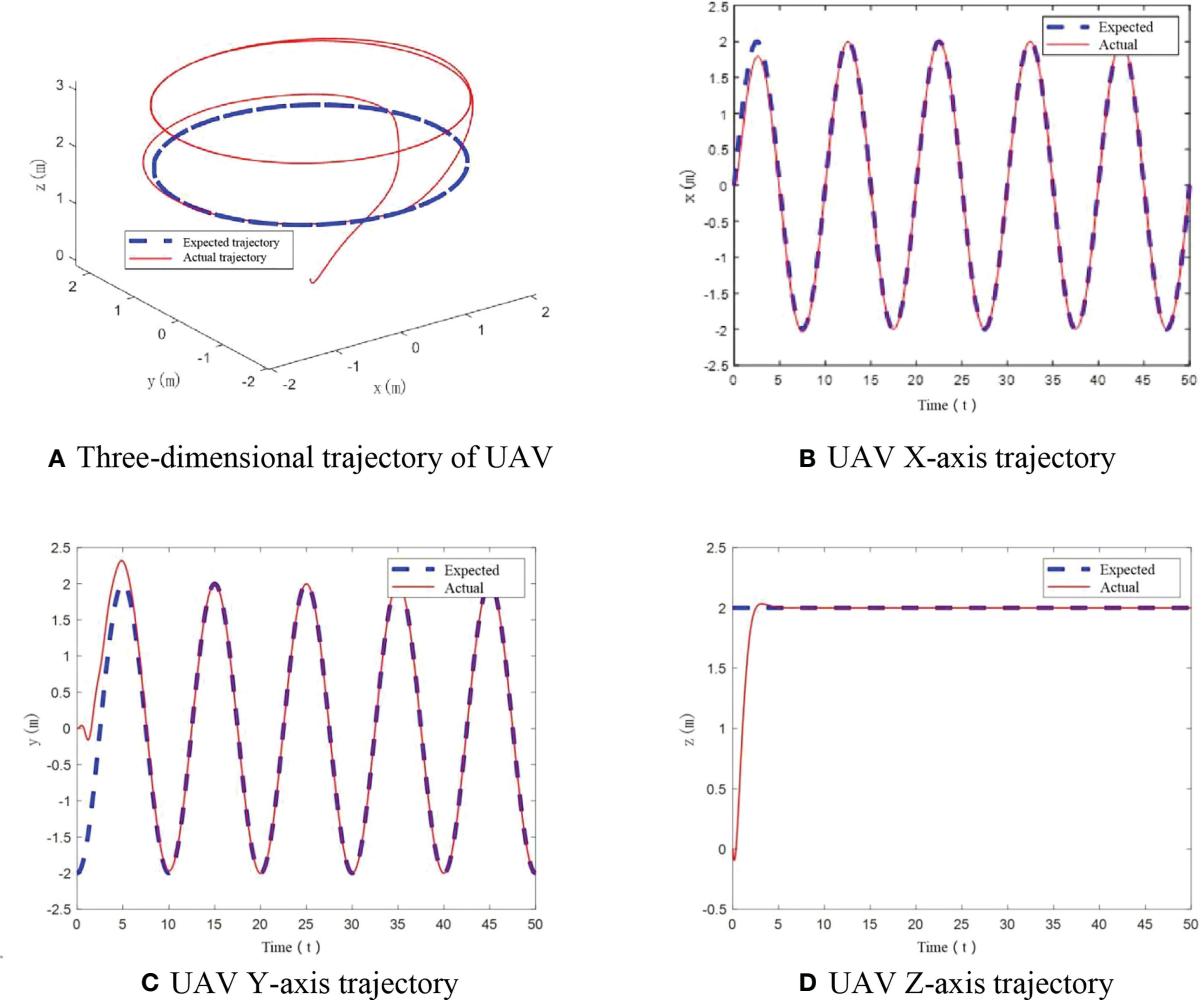
UAV flight trajectory with no mass change. **(A) **Three-dimensional trajectory of UAV, **(B)** UAV X-axis trajectory, **(C)** UAV Y-axis trajectory, **(D)** UAV Z-axis trajectory.

In this scenario, the UAV mass is 1.121 kg. The flight trajectory of UAV is shown in **Figures 5A–D**. From these diagrams, we can see that UAV can track the expected flight trajectory well under the designed control algorithm, which reflects good control performance. There is no obvious deviation in the whole, and the real-time tracking of the flight trajectory is realized.

**Figure 6:**
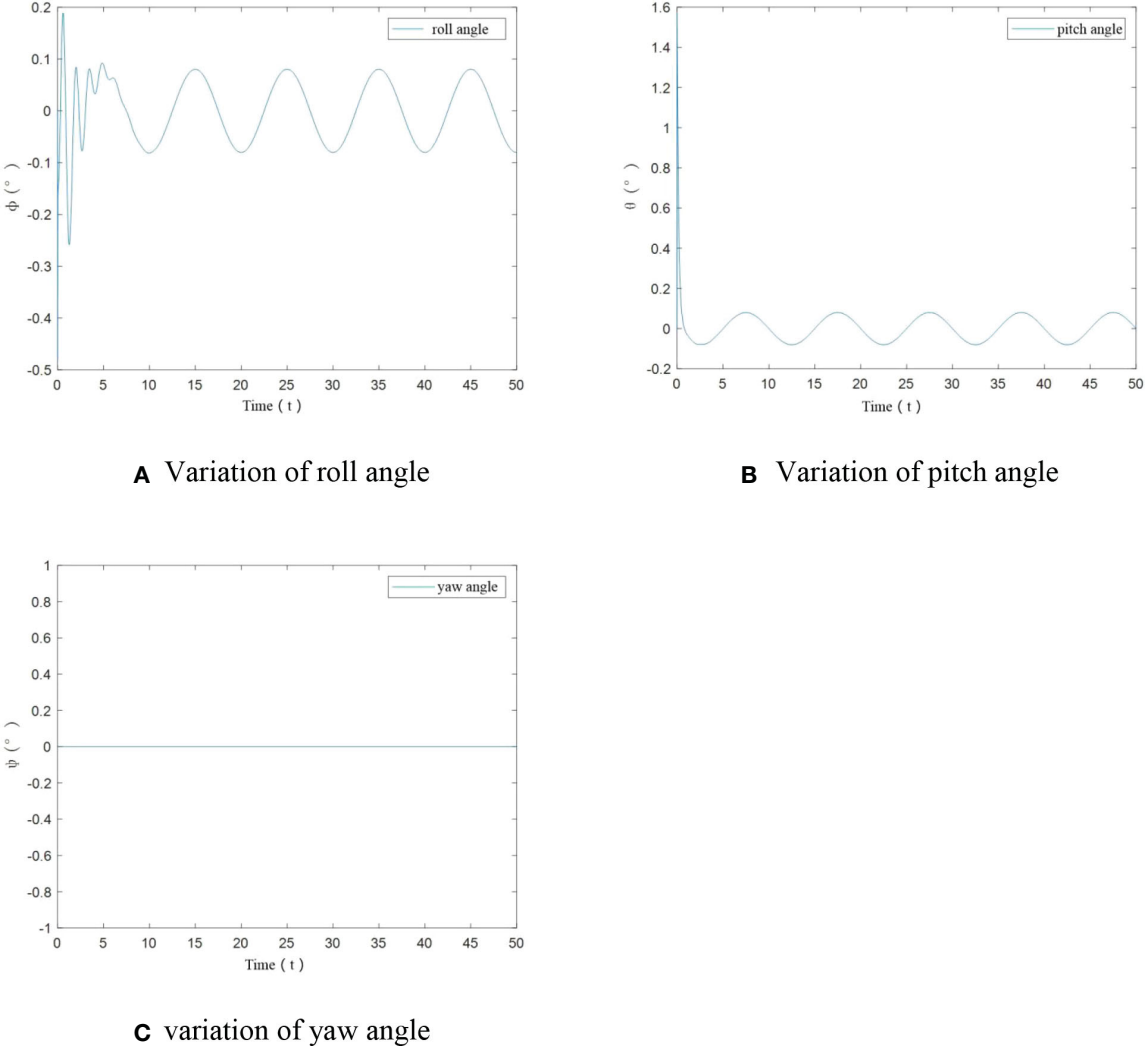
Angle variation of UAV under no-load condition. **(A)** Variation of roll angle, **(B)** Variation of pitch angle, **(C)** variation of yaw angle.

To further verify tracking performance of UAV under the designed control algorithm, angle variation diagrams are obtained. From **Figures 6A–C**, it can be seen that in this case, only at the start, roll angle and pitch angle have an angular change. After stabilization change of roll angle and pitch angle is less than ±0.1°. The yaw angle has been maintained at 0°. The designed control algorithm achieves the stability of UAV flight trajectory.

### 4.2 Small increase in UAV mass

**Figure 7:**
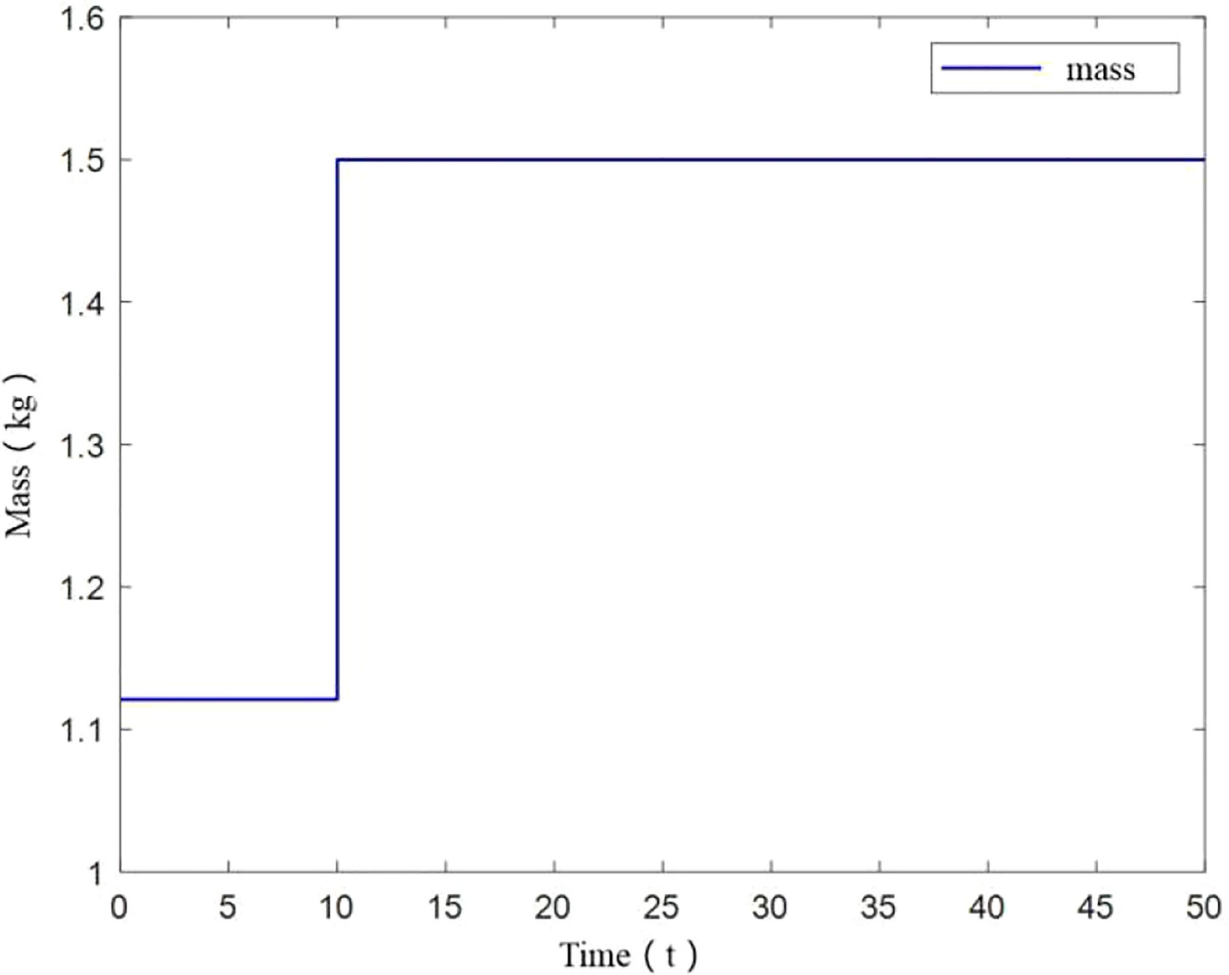
Mass change of UAV.

Under this simulation scenario, UAV mass is initially 1.121kg, and changes abruptly to 1.5kg at 10s. The mass change diagram is shown in **Figure 7**.

**Figure 8:**
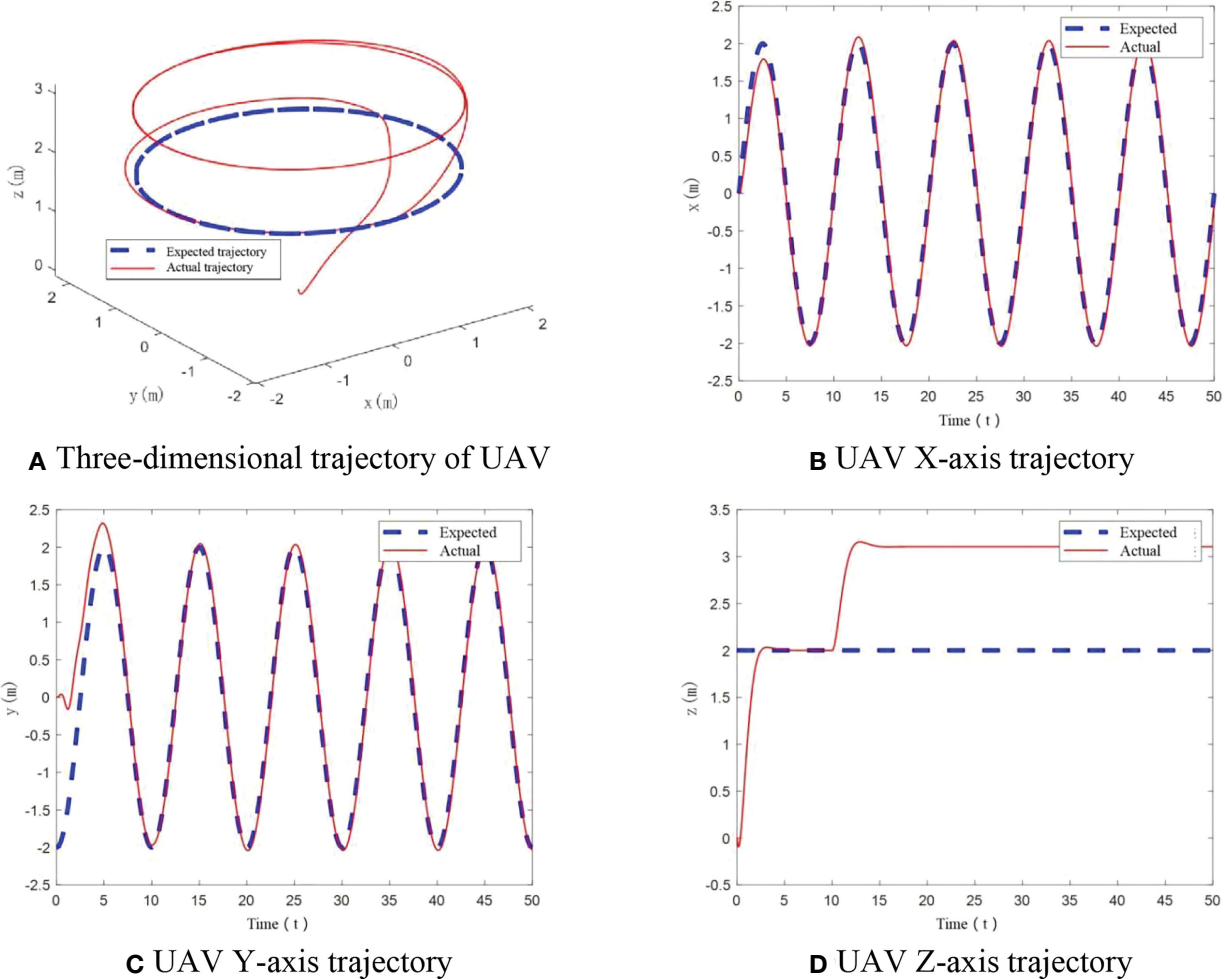
UAV flight trajectory with small mass change. **(A)** Three-dimensional trajectory of UAV, **(B)** UAV X-axis trajectory, **(C)** UAV Y-axis trajectory, **(D)** UAV Z-axis trajectory.

From **Figures 8B–D**, it can be seen that UAV tracks the desired trajectory well in X and Y axis with small mass increase, consistent with constant UAV mass. While in the vertical direction, a tracking error is generated, but eventually remains smooth. The angle variation diagram is obtained from **Figure 8**.

It should be noted that in **Figure 8D**, when UAV mass changes at small scale at 10s, z-axis trajectory deviates from the desired trajectory at 10-15s. And it does not return to the desired trajectory after the deviation. Mass variation has an influence on z-axis trajectory. So the control method is affected by change of load.

**Figure 9:**
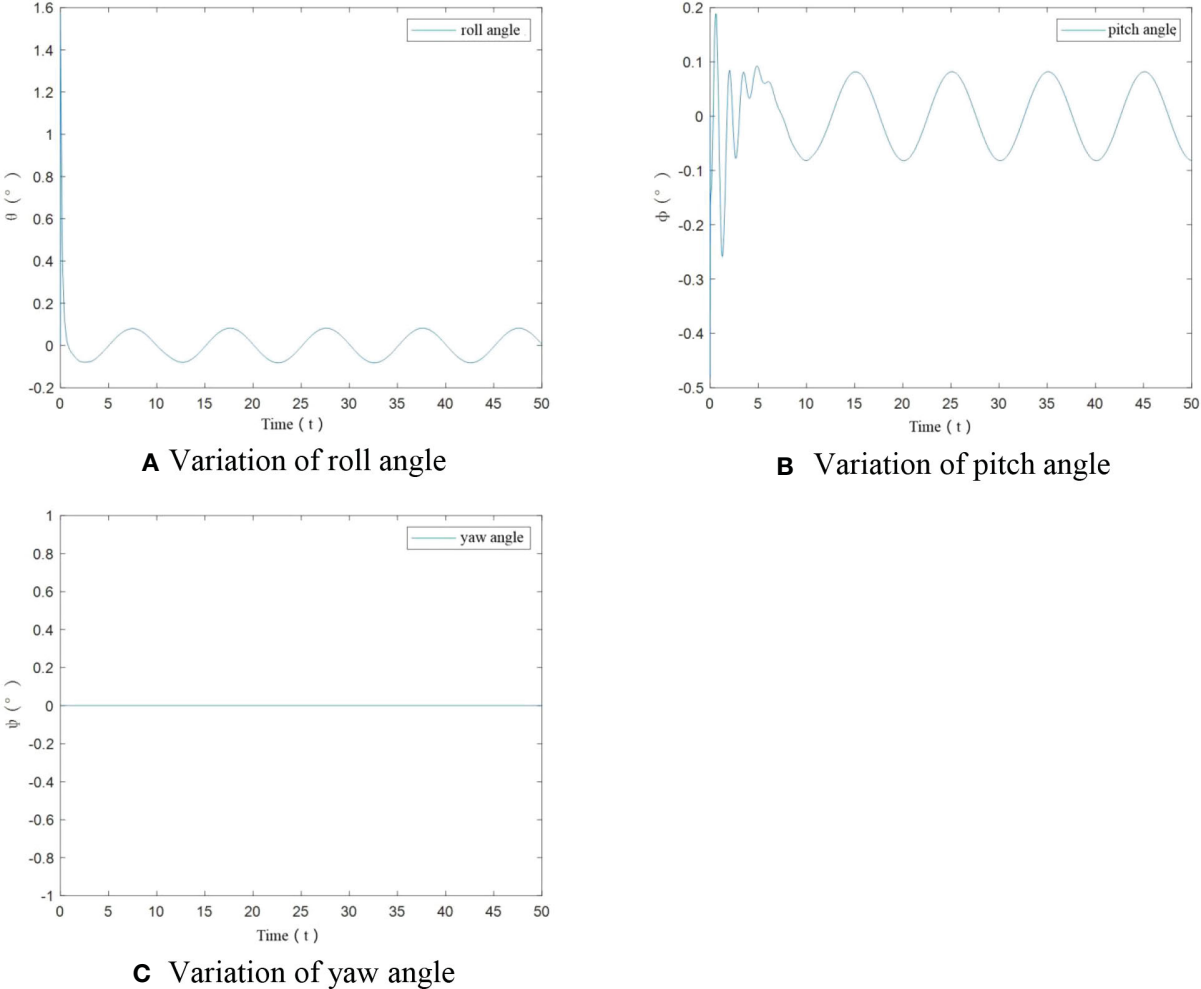
Angle variation of agricultural UAV under small load. **(A)** Variation of roll angle, **(B)** Variation of pitch angle, **(C)** Variation of yaw angle.

From **Figures 9A–C**, it can be seen that when small increase of UAV mass happens, the roll angle and pitch angle have an angular change only at start-up. After stabilization the change is less than ±0.1°. The yaw angle has been maintained at 0°. The algorithm can realize control of UAV flight trajectory stability.

### 4.3 Significant increase in UAV mass

**Figure 10:**
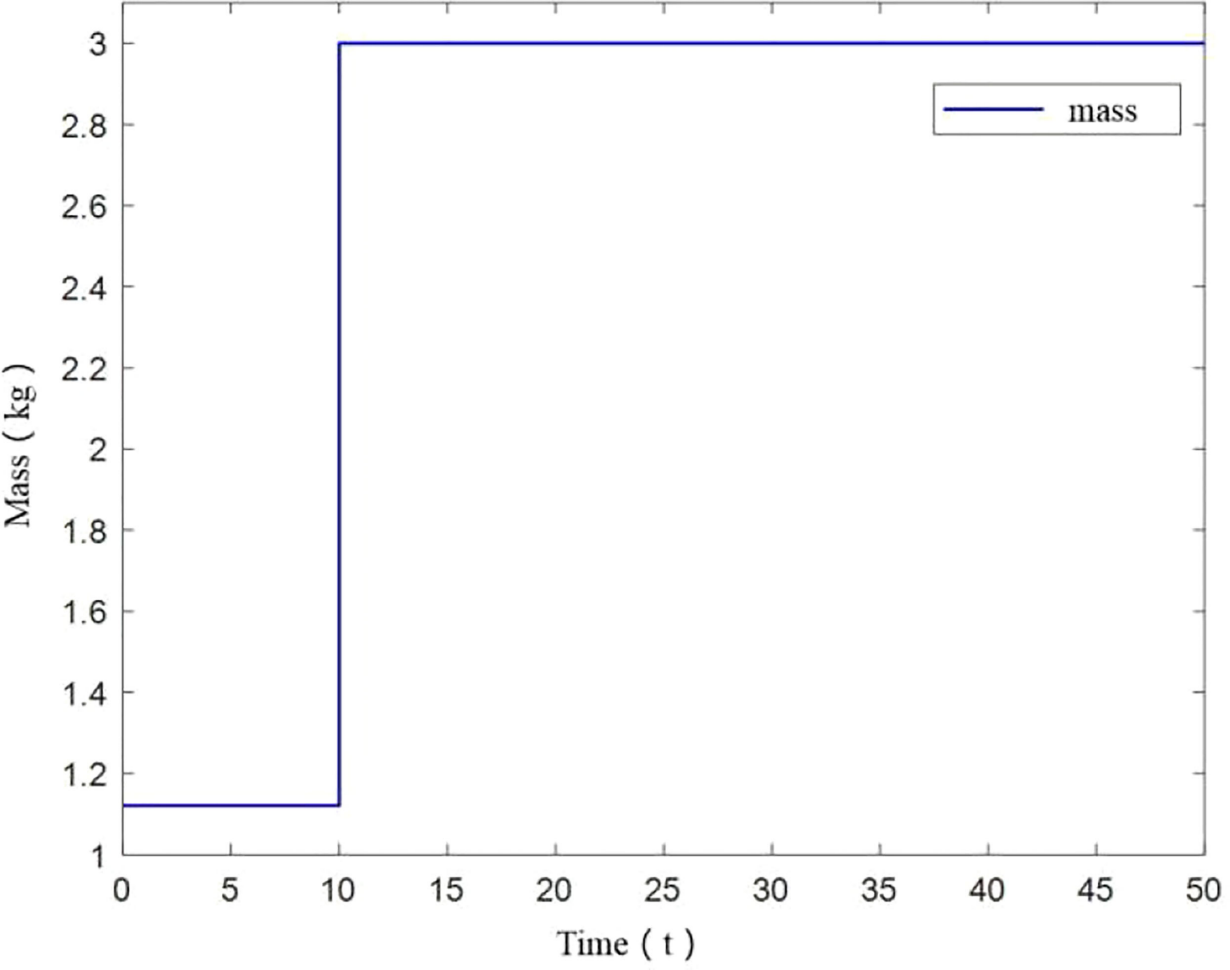
Significant change of UAV mass.

Considering a significant increase in UAV mass, the mass of UAV is also initially set to 1.121kg, which makes a significant increase to 1.5kg at 10s, indicating that a perturbation of the load occurs. The mass change diagram is shown in **Figure 10**.

**Figure 11:**
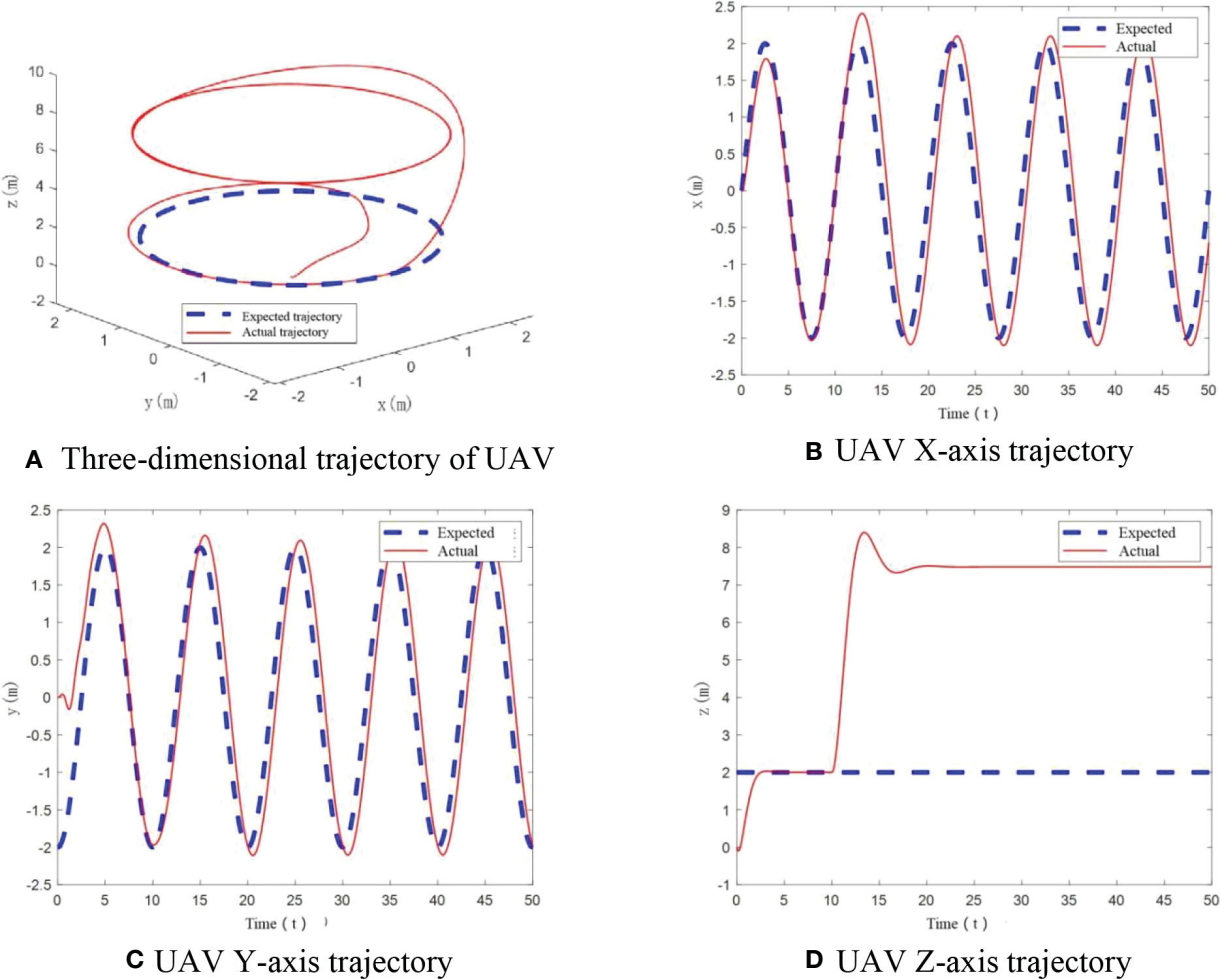
UAV flight trajectory with significant mass change. **(A)** Three-dimensional trajectory of UAV, **(B)** UAV X-axis trajectory, **(C)** UAV Y-axis trajectory, **(D)** UAV Z-axis trajectory.

The simulation gives flight trajectory as in **Figure 11A**. As can be seen in **Figure 11B–D**, when a significant increase in UAV mass happens, the UAV shows tracking errors in X and Y axis, which cannot track the desired trajectory well comparing to cases of constant UAV mass and small increase in UAV mass. In the vertical direction, the tracking error is further enlarged in comparison with the case of small UAV mass increase, and overshoot is more obvious.

**Figure 12:**
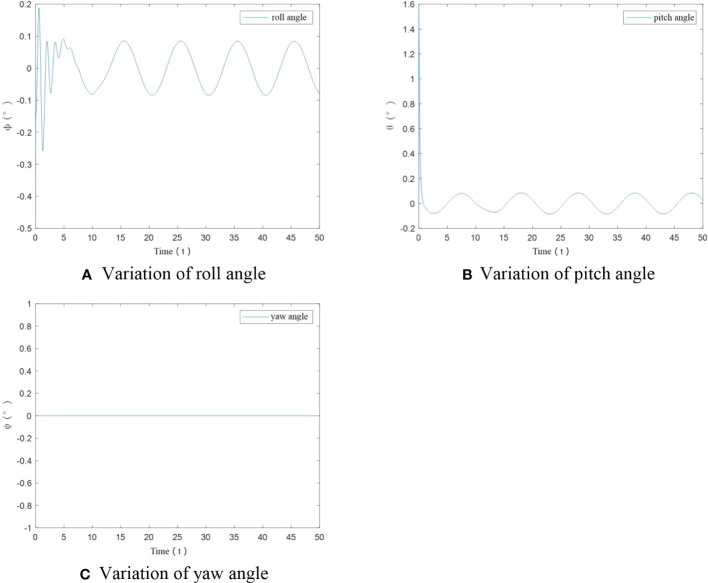
Angle variation of UAV under significant load.

From **Figure 12A–C**, it can be seen that roll angle and pitch angle have an angular change situation only at start-up when UAV mass increases significantly. After stabilization, the change in roll angle and pitch angle is less than ±0.1°, and the yaw angle has been maintained at 0°. The designed controller can realize the flight trajectory stability under significant load.

### 4.4 UAV mass change with time

**Figure 13:**
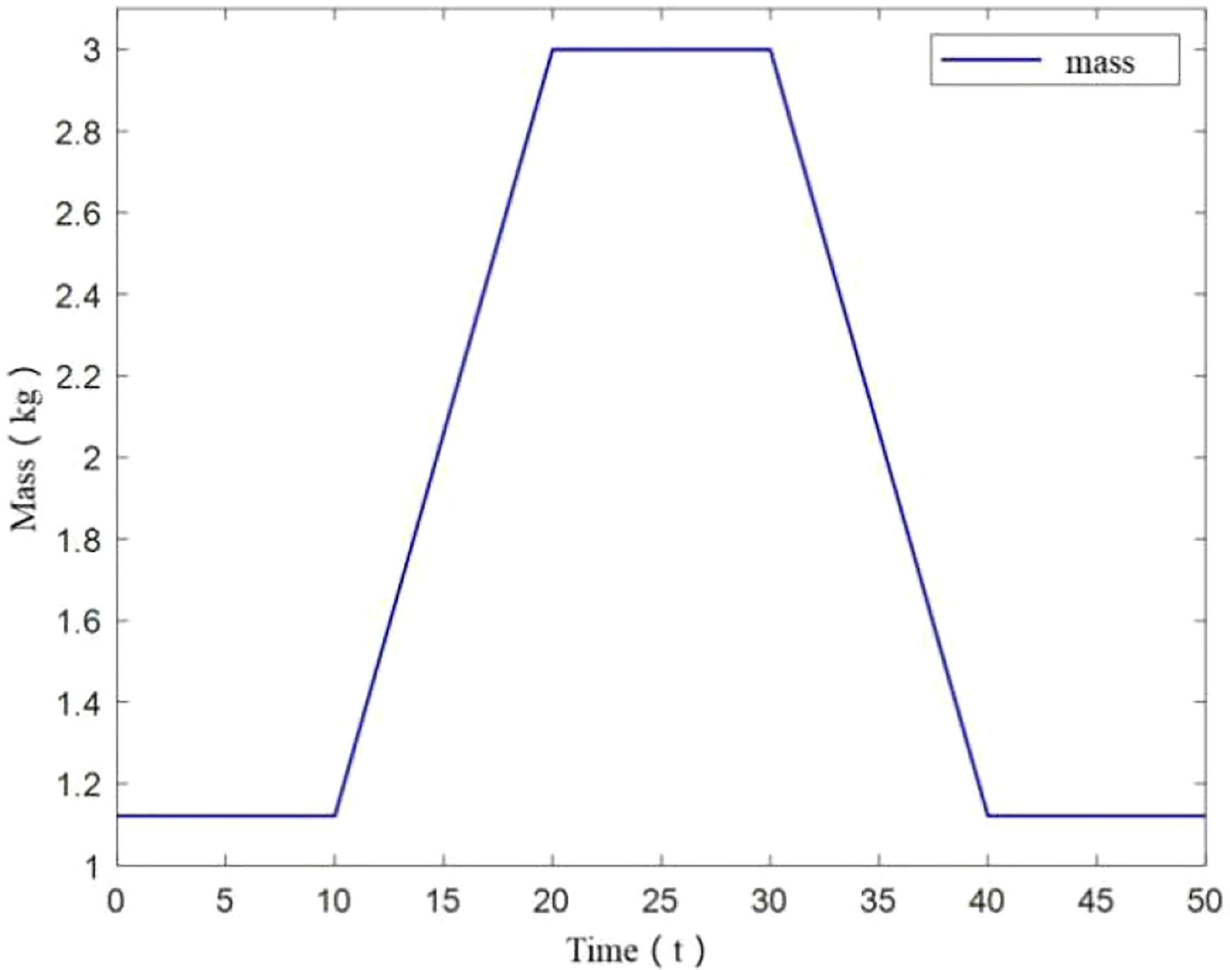
Mass change of UAV.

To better reflect effect of load perturbation, UAV mass was designed to change with time. In this scenario, UAV mass is initially 1.121kg, then it increases to 3kg from 10s to 20s. And this weight is maintained until 30s, after which the mass starts to decrease and reaches the initial mass at 40s as shown in **Figure 13**.

**Figure 14:**
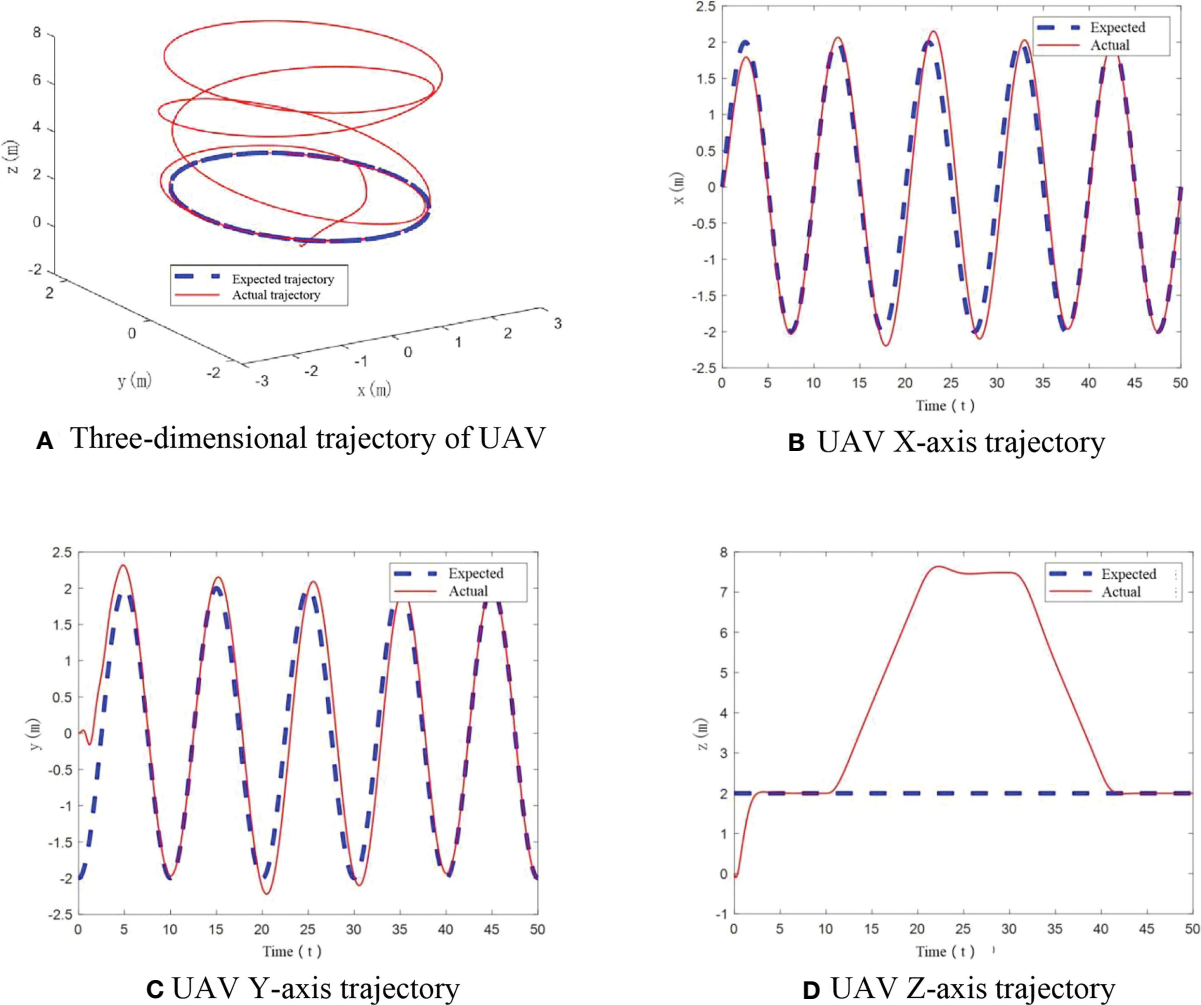
UAV flight trajectory with mass change. **(A)** Three-dimensional trajectory of UAV, **(B)** UAV X-axis trajectory, **(C)** UAV Y-axis trajectory, **(D)** UAV Z-axis trajectory.

This case better represents the effect of load perturbation on the stability of UAV flight trajectory, the perturbation shows dynamic changes with time, and this is closer to the real situation. The trajectory in this case is shown in **Figure 14A**.

From **Figures 14B–D**, it can be seen that under the condition of time-varying UAV mass, the trajectory of the UAV in X and Y axis can track the desired trajectory well when the mass does not vary much. The tracking error appears when the mass increases more. In the vertical direction, the tracking error of the UAV varies with the UAV mass change.

**Figure 15:**
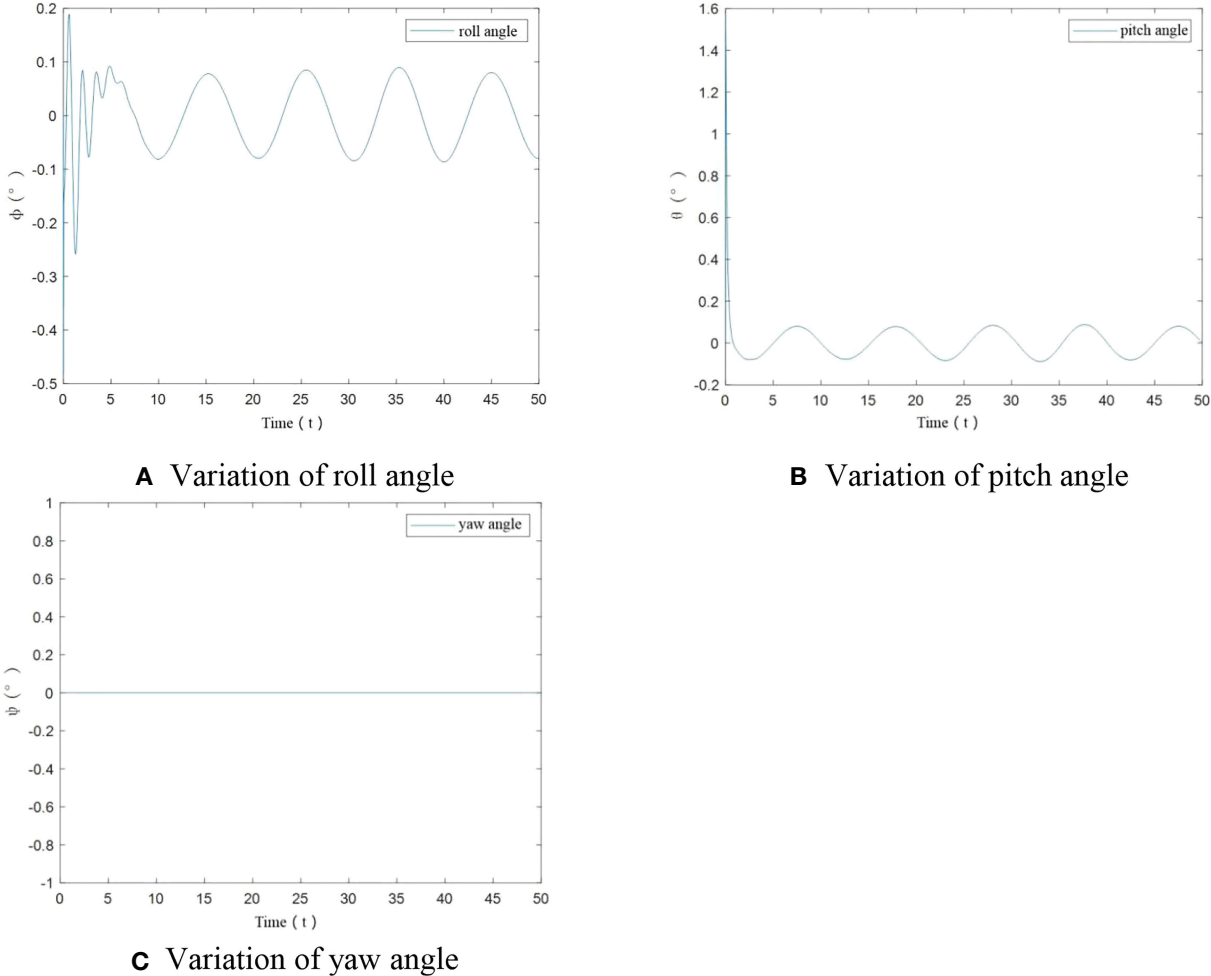
Angular variation for UAV under load variation. **(A)** Variation of roll angle, **(B)** Variation of pitch angle, **(C)** Variation of yaw angle.

From **Figures 15A–C**, it can be seen that roll angle and pitch angle have an angular change only at start-up. After stabilization, the change in roll angle and pitch angle is less than ±0.1°. The yaw angle has been maintained at 0°. The designed controller can realize the flight trajectory stabilization.

According to the simulation results, when the UAV has a significant change in mass, i.e., a large load, the UAV will have a significant error in tracking the desired trajectory, especially in the vertical axis direction.

## 5 Conclusion

In the scenario of small increase in mass, UAV can track the desired trajectory well in X and Y axis, and a tracking error is generated in the vertical direction, but it eventually remains steady. In the scenario of a significant increase in mass, UAV tracked the desired trajectory tracking in X and Y axis, with a bigger tracking error in the vertical direction. In the scenario of time-varying mass, the UAV tracked the desired trajectory well in X and Y axis when the mass did not change much, and tracking errors appeared when the mass increased more. In vertical direction, the tracking error varies with the mass of the UAV.

In summary, taking agricultural UAV as research object, we adopt robust T-S fuzzy control method in attitude angle control part and PID controller in position loop control part to make UAV flight trajectory achieve stability in the simulation environment of load variation. Due to the sudden change of mass, the trajectory of UAV will deviate from the expected path, but its tracking error is small, so that the designed controller can effectively solve the trajectory tracking problem of small variable load against perturbation.

## Data availability statement

The original contributions presented in the study are included in the article/supplementary material. Further inquiries can be directed to the corresponding author.

## Author contributions

XW: Conceptualization, Methodology, Supervision. WX: Software, Validation. LJ: Writing - Original Draft, Data Curation. YY: Formal analysis, Writing - Review & Editing. All authors contributed to the article and approved the submitted version.
